# Supplementary material for: The Antimicrobial Activity of Cannabinoids
Source: Antibiotics (Basel). 2020 Jul 13;9(7):406. doi: 10.3390/antibiotics9070406 (PMC7400265; doi:10.3390/antibiotics9070406)
Supplement: Supplementary file 1 [file antibiotics-09-00406-s001.pdf]

Supporting Information

Antimicrobial Structure-activity Relationships of Cannabinoids

John A. Karas, Labell J. M. Wong, Olivia K. A. Paulin, Amna C. Mazeh, Maytham H. Hussein, Jian Li, Tony Velkov*

Antibacterial activity of Δ^{9}-tetrahydrocannabinol and cannabidiol
van Klingeren, B., Ham, M. T.,
Antonie Van Leeuwenhoek, **1976**, 42, 9-12.

MIC reported in μg/mL

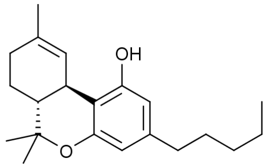

1. Δ^{9}-tetrahydrocannabinol
Nutrient broth agar
S. aureus (4 strains) = 2-5
S. pyogenes (1 strain) = 5
S. milleri (1 strain) = 2
S. faecalis (1 strain) = 5
E. coli (4 strains) > 100
S. typhi (1 strain) > 100
P. vulgaris (1 strain) > 100
Horse blood agar
S. aureus (4 strains) = 20-50
S. pyogenes (1 strain) = 50
S. milleri (1 strain) = 50

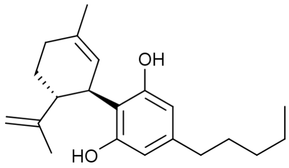

2. Cannabidiol
Nutrient broth agar
S. aureus (4 strains) = 1-5
S. pyogenes (1 strain) = 2
S. milleri (1 strain) = 1
S. faecalis (1 strain) = 5
E. coli (4 strains) > 100
S. typhi (1 strain) > 100
P. vulgaris (1 strain) > 100
Horse blood agar
S. aureus (4 strains) = 20-50
S. pyogenes (1 strain) = 50
S. milleri (1 strain) = 50

Biological Activity of Cannabichromene, its Homologs and Isomers
Turner, C. E., Elsohly, M. A.,
J. Clin. Pharmacol., **1981**, 21, 283S−291S.

MIC reported in µg/mL

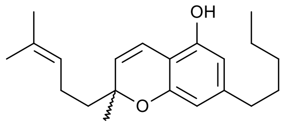

<u>**3.** Cannabichromene (IV)</u>
B. subtilis 24 h = 0.39
B. subtilis 48 h = 0.78
S. aureus 24 h = 1.56
S. aureus 48 h = 1.56
M. smegmatis 24 h = 12.5
M. smegmatis 48 h = 25.0
C. albicans 48 h = NT
C. albicans 72 h = NT
S. cerevisiae 48 h = 25
S. cerevisiae 72 h = 50
T. mentagrophytes 48 h = 25
T. mentagrophytes 72 h = 50

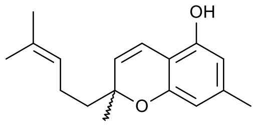

<u>**4.** Cannabichromene-C_1 (V)</u>
B. subtilis 24 h = 3.12
B. subtilis 48 h = 3.12
S. aureus 24 h = 3.12
S. aureus 48 h = 3.12
M. smegmatis 24 h = 3.12
M. smegmatis 48 h = 6.25
C. albicans 48 h = NT
C. albicans 72 h = NT
S. cerevisiae 48 h = 6.25
S. cerevisiae 72 h = 12.5
T. mentagrophytes 48 h = 6.25
T. mentagrophytes 72 h = 6.25

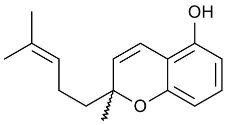

<u>**5.** Cannabichromene-C_0 (VI)</u>
B. subtilis 24 h = 6.25
B. subtilis 48 h = 12.5
S. aureus 24 h = 12.5
S. aureus 48 h = 12.5
M. smegmatis 24 h = 12.5
M. smegmatis 48 h = 12.5
C. albicans 48 h = 50
C. albicans 72 h = 50
S. cerevisiae 48 h = 25
S. cerevisiae 72 h = 25
T. mentagrophytes 48 h = 25
T. mentagrophytes 72 h = 25

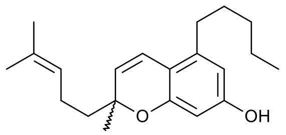

<u>**6.** Isocannabichromene (VII)</u>
B. subtilis 24 h = 0.78
B. subtilis 48 h = 3.12
S. aureus 24 h = NT
S. aureus 48 h = NT
M. smegmatis 24 h = 25.0
M. smegmatis 48 h = 25.0
C. albicans 48 h = 50
C. albicans 72 h = 100
S. cerevisiae 48 h = NT
S. cerevisiae 72 h = NT
T. mentagrophytes 48 h = NT
T. mentagrophytes 72 h = NT

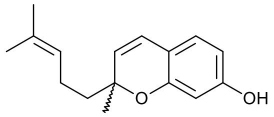

<u>**7.** Isocannabichromene-C_0 (VIII)</u>
B. subtilis 24 h = 6.25
B. subtilis 48 h = 6.25
S. aureus 24 h = 12.5
S. aureus 48 h = 12.5
M. smegmatis 24 h = 12.5
M. smegmatis 48 h = 12.5
C. albicans 48 h = 12.5
C. albicans 72 h = 25
S. cerevisiae 48 h = NT
S. cerevisiae 72 h = NT
T. mentagrophytes 48 h = 6.25
T. mentagrophytes 72 h = 6.25

Antibacterial Cannabinoids from *Cannabis sativa*: A Structure-Activity Study

Appendino, G., Gibbons, S., Giana, A., Pagani, A., Grassi, G., Stavri, M.,
Smith, E., Mukhlesur Rahman, M.,
J. Nat. Prod., **2008**, 71, 1427–1430.

MIC reported in µg/mL

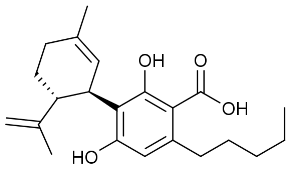

8. Analog 1a
S. aureus SA-1199B = 2
S. aureus RN-4220 = 2
S. aureus XU212 = 2
S. aureus ATCC25923 = 2
S. aureus EMRSA-15 = 2
S. aureus EMRSA-16 = 2

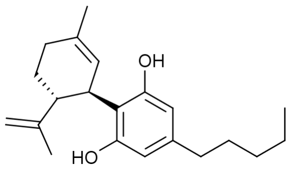

9. Analog 1b
S. aureus SA-1199B = 1
S. aureus RN-4220 = 1
S. aureus XU212 = 1
S. aureus ATCC25923 = 0.5
S. aureus EMRSA-15 = 1
S. aureus EMRSA-16 = 1

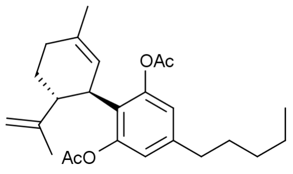

10. Analog 1c
S. aureus SA-1199B > 128
S. aureus RN-4220 > 128
S. aureus XU212 > 128
S. aureus ATCC25923 > 128
S. aureus EMRSA-15 > 128
S. aureus EMRSA-16 > 128

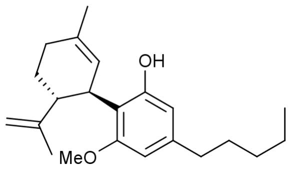

11. Analog 1d
S. aureus SA-1199B > 128
S. aureus RN-4220 > 128
S. aureus XU212 > 128
S. aureus ATCC25923 > 128
S. aureus EMRSA-15 > 128
S. aureus EMRSA-16 > 128

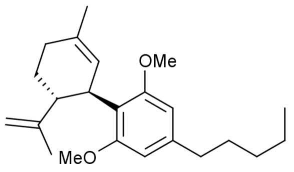

12. Analog 1e
S. aureus SA-1199B > 128
S. aureus RN-4220 > 128
S. aureus XU212 > 128
S. aureus ATCC25923 > 128
S. aureus EMRSA-15 > 128
S. aureus EMRSA-16 > 128

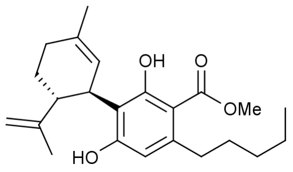

13. Analog 1f
S. aureus SA-1199B > 128
S. aureus RN-4220 > 128
S. aureus XU212 > 128
S. aureus ATCC25923 > 128
S. aureus EMRSA-15 > 128
S. aureus EMRSA-16 > 128

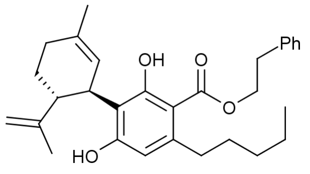

14. Analog 1g
S. aureus SA-1199B > 128
S. aureus RN-4220 > 128
S. aureus XU212 = > 128
S. aureus ATCC25923 > 128
S. aureus EMRSA-15 > 128
S. aureus EMRSA-16 > 128

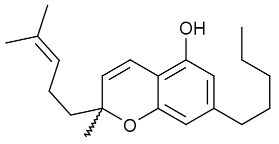

15. Analog 2
S. aureus SA-1199B = 2
S. aureus RN-4220 = 2
S. aureus XU212 = 1
S. aureus ATCC25923 = 2
S. aureus EMRSA-15 = 2
S. aureus EMRSA-16 = 2

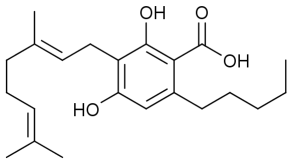

16. Analog 3a
S. aureus SA-1199B = 4
S. aureus RN-4220 = 2
S. aureus XU212 = 4
S. aureus ATCC25923 = 4
S. aureus EMRSA-15 = 2
S. aureus EMRSA-16 = 4

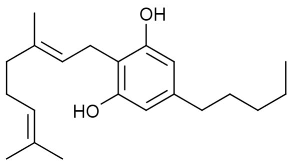

17. Analog 3b
S. aureus SA-1199B = 1
S. aureus RN-4220 = 1
S. aureus XU212 = 1
S. aureus ATCC25923 = 1
S. aureus EMRSA-15 = 2
S. aureus EMRSA-16 = 1

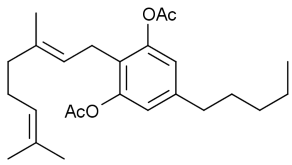

18. Analog 3c
S. aureus SA-1199B > 128
S. aureus RN-4220 > 128
S. aureus XU212 > 128
S. aureus ATCC25923 > 128
S. aureus EMRSA-15 > 128
S. aureus EMRSA-16 > 128

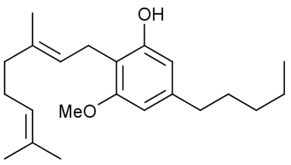

19. Analog 3d
S. aureus SA-1199B > 128
S. aureus RN-4220 > 128
S. aureus XU212 > 128
S. aureus ATCC25923 > 128
S. aureus EMRSA-15 > 128
S. aureus EMRSA-16 > 128

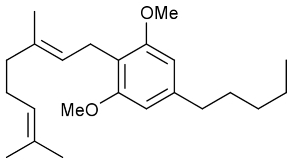

20. Analog 3e
S. aureus SA-1199B > 128
S. aureus RN-4220 > 128
S. aureus XU212 > 128
S. aureus ATCC25923 > 128
S. aureus EMRSA-15 > 128
S. aureus EMRSA-16 > 128

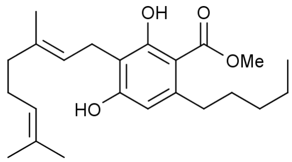

21. Analog 3f
S. aureus SA-1199B = 64
S. aureus RN-4220 = ND
S. aureus XU212 = 64
S. aureus ATCC25923 = ND
S. aureus EMRSA-15 = ND
S. aureus EMRSA-16 = ND

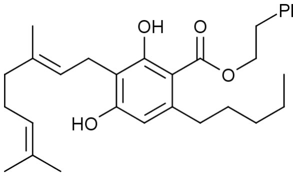

22. Analog 3g
S. aureus SA-1199B > 128
S. aureus RN-4220 > 128
S. aureus XU212 > 128
S. aureus ATCC25923 > 128
S. aureus EMRSA-15 > 128
S. aureus EMRSA-16 > 128

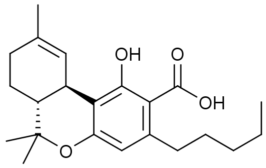

23. Analog 4a
S. aureus SA-1199B = 8
S. aureus RN-4220 = 4
S. aureus XU212 = 8
S. aureus ATCC25923 = 4
S. aureus EMRSA-15 = 8
S. aureus EMRSA-16 = 4

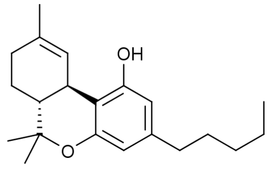

24. Analog 4b
S. aureus SA-1199B = 2
S. aureus RN-4220 = 1
S. aureus XU212 = 1
S. aureus ATCC25923 = 1
S. aureus EMRSA-15 = 2
S. aureus EMRSA-16 = 0.5

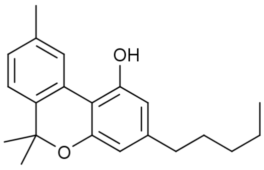

25. Analog 5
S. aureus SA-1199B = 1
S. aureus RN-4220 = 1
S. aureus XU212 = 1
S. aureus ATCC25923 = 1
S. aureus EMRSA-15 = 1
S. aureus EMRSA-16 = ND

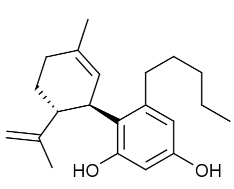

26. Analog 6
S. aureus SA-1199B = 1
S. aureus RN-4220 = 1
S. aureus XU212 = 1
S. aureus ATCC25923 = 1
S. aureus EMRSA-15 = 1
S. aureus EMRSA-16 = 1

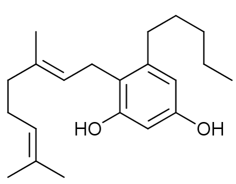

27. Analog 7
S. aureus SA-1199B = 2
S. aureus RN-4220 = 1
S. aureus XU212 = 0.5
S. aureus ATCC25923 = 1
S. aureus EMRSA-15 = 2
S. aureus EMRSA-16 = ND

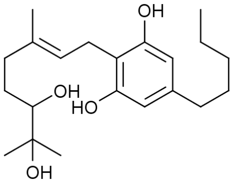

28. Analog 8
S. aureus SA-1199B = 32
S. aureus RN-4220 = 32
S. aureus XU212 = 16
S. aureus ATCC25923 = 16
S. aureus EMRSA-15 = 16
S. aureus EMRSA-16 = 32

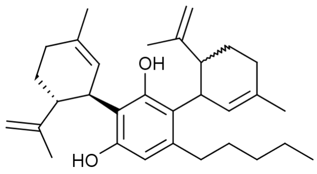

29. <u>Analog 9</u>
S. aureus SA-1199B > 128
S. aureus RN-4220 > 128
S. aureus XU212 > 128
S. aureus ATCC25923 > 128
S. aureus EMRSA-15 > 128
S. aureus EMRSA-16 > 128

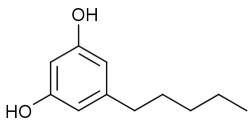

30. <u>Analog 10</u>
S. aureus SA-1199B = 64
S. aureus RN-4220 = 64
S. aureus XU212 = 64
S. aureus ATCC25923 = 128
S. aureus EMRSA-15 = 64
S. aureus EMRSA-16 = 64

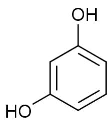

31. <u>Analog 11</u>
S. aureus SA-1199B > 256
S. aureus RN-4220 > 256
S. aureus XU212 > 256
S. aureus ATCC25923 > 256
S. aureus EMRSA-15 > 256
S. aureus EMRSA-16 > 256

Antimicrobial potential of endocannabinoid and endocannabinoid-likecompounds against methicillin-resistant *Staphylococcus aureus*
Feldman, M., Smoum, R., Mechoulam, R., Steinberg, D.,
Sci. Rep., **2018**, 8, 17696.

MIC reported in µg/mL

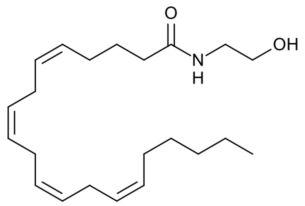

32. <u>Anandamide</u>
MRSA 33592 > 256
MRSA CI > 256
MRSA 43300 > 256

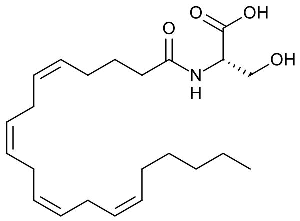

33. <u>Arachidonoyl serine</u>
MRSA 33592 = 32
MRSA CI > 256
MRSA 43300 128

Cannabidiol is an effective helper compound in combination with bacitracin to kill Gram-positive bacteria
Wassmann, C. S., Højrup, P., Klitgaard, J. K.,
Sci. Rep., **2020** 10, 4112.

MIC reported in µg/mL

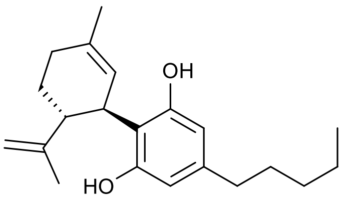

34. Cannabidiol
MRSA USA300 FPR3757 = 4
E. faecalis (13–327129) = 8
L. monocytogenes (EGD) = 4
MRSE (933010 3F-16 b4) = 4

Uncovering the Hidden Antibiotic Potential of Cannabis
Farha, M. A., El-Halfawy, O. M., Gale R. T., MacNair, C. R., Carfrae, L. A., Zhang, X., Jentsch, N. G., Magolan, J., Brown, E. D.,
ACS Infect. Dis., **2020**, 6, 338−346.

MIC reported in µg/mL

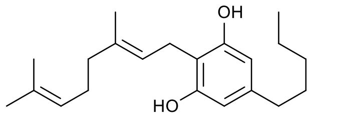

35. Cannabigerol
MRSA USA300 = 2
E. coli > 128

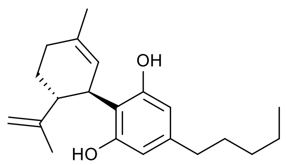

36. Cannabidiol
MRSA USA300 = 2
E. coli > 128

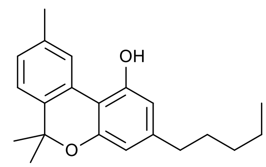

37. Cannabinol
MRSA USA300 = 2
E. coli > 128

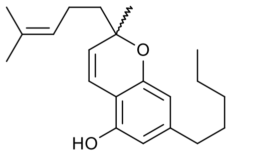

38. Cannabichromene
MRSA USA300 = 8
E. coli > 128

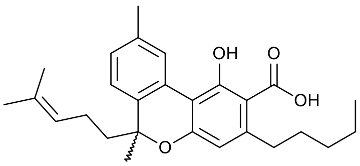

39. Cannabichromenic acid
MRSA USA300 = 2
E. coli > 128

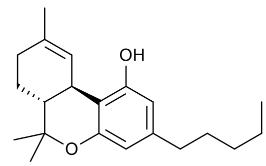

40. (-)Δ^9-Tetrahydrocannabinol
MRSA USA300 = 2
E. coli > 128

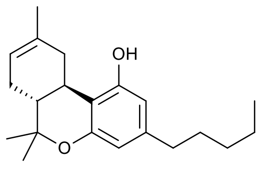

41. (-)Δ^8-Tetrahydrocannabinol
MRSA USA300 = 2
E. coli > 128

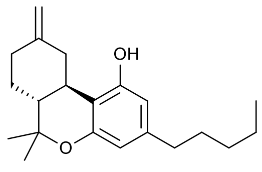

42. *exo*-Tetrahydrocannabinol
MRSA USA300 = 2
E. coli > 128

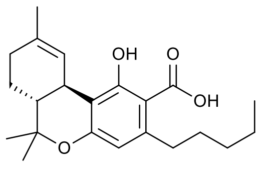

43. (-)Δ^9-Tetrahydrocannabinol acid A
MRSA USA300 = 4
E. coli > 128

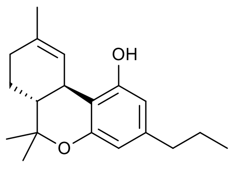

44. Δ^9-Tetrahydrocannabivarin
MRSA USA300 = 4
E. coli > 128

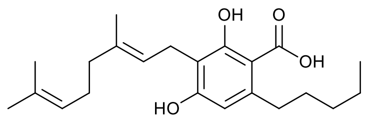

45. Cannabigerolic acid
MRSA USA300 = 4
E. coli > 128

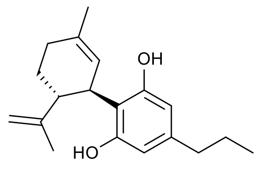

46. Cannabidivarin
MRSA USA300 = 8
E. coli > 128

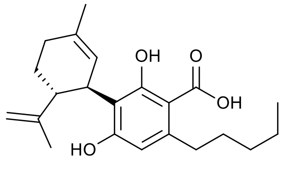

47. Cannabidiolic acid
MRSA USA300 = 16
E. coli > 128

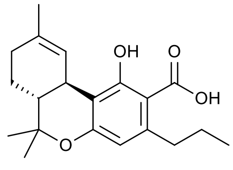

48. Tetrahydrocannabivarinic acid
MRSA USA300 = 16
E. coli > 128

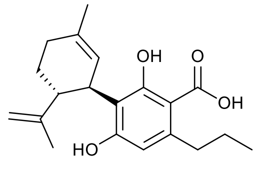

49. Cannabidivarinic acid
MRSA USA300 = 32
E. coli > 128

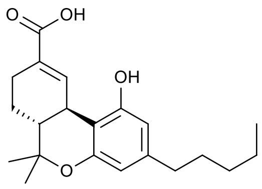

50. (±) 11-nor-9-carboxy-Δ^9-THC
MRSA USA300 > 32
E. coli > 128

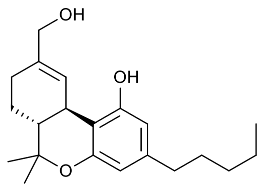

51. (±) 11-hydroxy-Δ^9-THC
MRSA USA300 > 32
E. coli > 128

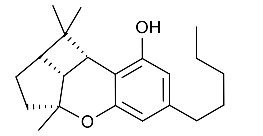

52. Cannabicyclol
MRSA USA300 > 32
E. coli > 128
